# Supplementary material for: Convergence between the microcosms of Southeast Asian and North American pitcher plants
Source: eLife. 2018 Aug 28;7:e36741. doi: 10.7554/eLife.36741 (PMC6130972; doi:10.7554/eLife.36741)
Supplement: Supplementary file 1. [file elife-36741-supp1.docx]

**Supplementary Information**

**See excel file for Dataset S1: Metadata**

**SI Tables:**

| **Table S1: Species information** | | | | | | | | | | |
| --- | --- | --- | --- | --- | --- | --- | --- | --- | --- | --- |
| Genus | Species | Loc. | Collection sites | No. samples | Avg. pH (+/- SD) | Avg. volume, mL (+/-SD) | Form | Avg. width, cm (+/-SD) | Avg. height, cm (+/-SD) | Aspect ratio |
| *Nepenthes* | *amp.* | Sing. | KRP, BTNP, UPR | 37 | 4.7 (0.5) | 8.0 (8.5) | squat | 9.0 (2.8) | 13.0 (4.2) | 0.69 |
|  | *gracilis* | Sing. | KRP, BTNP, UPR | 35 | 3.2 (1.4) | 2.4 (1.3) | squat | 3.0 (NA) | 11.0 (NA) | 0.27 |
|  | *hirsuta* | MB | NG | 16 | 5.1 (0.6) | 5.3 (3.2) | squat | 7.0 (1.4) | 16.5 (2.1) | 0.42 |
|  | *raff.* | Sing. | KRP, BTNP, UPR | 42 | 4.6 (1.4) | 10.1 (9.2) | squat | 14.0 (1.4) | 23.5 (2.1) | 0.60 |
|  | *rein.* | MB | NG | 12 | 4.7 (0.5) | 6.9 (2.9) | squat | 6.0 (1.4) | 22.5 (10.6) | 0.27 |
|  | *steno.* | MB | NG | 24 | 2.8 (1.5) | 15.6 (13.1) | squat | 6.7 (1.5) | 18.7 (5.5) | 0.36 |
|  | *tent.* | MB | NG | 8 | 5.4 (1.3) | 2.8 (2.2) | squat | 3.0 (0) | 13.5 (2.1) | 0.22 |
|  | *veitchii* | MB | NG | 19 | 4.4 (0.9) | 130 (171) | squat | 8.5 (3.5) | 26.5 (9.2) | 0.32 |
| *Sarracenia* | *alata* | GC | SB, BF, OFB, WB | 24 | .05 (1.2) | 2.7 (2.5) | tall | 4.0 (1.4) | 40.0 (28.3) | 0.10 |
|  | *flava* | GC | BW, NP, MS, CB, PPS, EB1 | 30 | 5.1 (1.0) | 6.0 (6) | tall | 6.0 (2.8) | 55.0 (21.2) | 0.11 |
|  | *leuco.* | GC | SH, BW, NB, EB1, EB2, WB | 35 | 5.5 (1.2) | 7.0 (7.6) | tall | 5.0 (1.4) | 55.0 (21.2) | 0.09 |
|  | *purp.* | MA | TS | 20 | 4.8 (0.8) | 11.1 (9.0) | squat | 3.0 (1.4) | 12.5 (3.5) | 0.24 |
|  | *rosea* | GC | SH, MS, CB, PPS | 23 | 5.6 (1.1) | 13.2 (7.9) | squat | 4.5 (2.1) | 17.5 (10.6) | 0.26 |
|  | *rubra* | GC | SH, EB1, EB2 | 11 | 6.1 (1.4) | 2.3 (3.6) | tall | 2.8 (1) | 25.5 (15.2) | 0.11 |
| Legend: *amp. = ampullaria; raff. = rafflesiana, rein; = reinwardtiana; steno. = stenophylla; tent. = tentaculata; lecuo. = leucophylla; purp. = purpurea*; Loc. = Location; Sing. = Singapore; MB = Maliau Basin; GC = Gulf Coast; MA = Massachusetts; KRP = Kent Ridge Park; BTNP = Bukit Timah Nature Preserve; UPR = between Upper and Lower Peirce Reservoir; NG = Nepenthes Garden; SB = Sweet Bay Bogs Preserve, MS; BF = Buttercup Flats, MS; OFB = Old Fort Bayou, MS; WB = Week's Bay Pitcher Plant Bog, AL; SH = Splinter Hill Bog, AL; BW = Blackwater River State Forest, FL; NP = Nokuse Plantation, FL; NB = Nokuse Bog, FL; MS =Mud Swamp New River Bog, FL ; CB = Crystal Bog, FL; PPS = Plea Phase Savanna, FL; EB1 = Eglin Airforce Base site 1, FL; EB2 = Eglin Airforce Base site 2, FL; TS = Tom Swamp, MA. | | | | | | | | | | |
|  |  |  |  |  |  |  |  |  |  |  |
|  |  |  |  |  |  |  |  |  |  |  |
|  |  |  |  |  |  |  |  |  |  |  |
|  |  |  |  |  |  |  |  |  |  |  |
|  |  |  |  |  |  |  |  |  |  |  |
|  |  |  |  |  |  |  |  |  |  |  |

**Table S2: Numbers of amplicon sequences and OTUs**

|  | **Primer set** | **16S** | **18S** |
| --- | --- | --- | --- |
|  | Samples | 492 | 472 |
| All data (post quality control) | Sequences | 12,375,647 | 14,664,713 |
|  | OTUs | 139,549 | 34,236 |
| Without observations of fewer than 10 sequences | Sequences | 11,337,226 | 14,447,905 |
|  | OTUs | 12,940 | 7,516 |

**Table S3**: **Shannon Diversity and Sample Volume**

|  | Whole dataset | | Subset | |
| --- | --- | --- | --- | --- |
| **Shannon Diversity** | Bacteria avg. (+/-SD) | Eukaryota avg. (+/-SD) | Bacteria avg. (+/-SD) | Eukaryota avg. (+/-SD) |
| Soil | 9.3 ( 0.7) | 5.5 (1.4) | 8.7 (0.9) | 5.0 (2.2) |
| Bog | 8.3 ( 0.9) | 5.2 (1.2) | 7.7 (1.0) | 5.0 (0.6) |
| *Nepenthes* | 5.2 (1.8) | 3.1 (0.9) | 5.5 (1.6) | 3.1 (1.0) |
| *Sarracenia* | 4.6 (1.2) | 2.2 (0.9) | 4.6 (1.0) | 2.1 (0.9) |
| **Sample Volume** |  |  |  |  |
| Soil | 7.0 ( 0) | 7.0 ( 0) | 7.0 (0) | 7.0 ( 0) |
| Bog | 7.4 ( 0.2) | 7.4 (0.2) | 7.4 (0.3) | 7.4 (0.3) |
| *Nepenthes* | 23.5 (71.8) | 23.1 (71.4) | 11.1 (15.2) | 10.9 (15.4) |
| *Sarracenia* | 7.3 (7.6) | 8.1 (7.8) | 6.2 (6.5) | 7.0 (6.8) |

| **Table S4. Comparing the Beta-diversity of communities** | | | |  | |  | |  | |  |
| --- | --- | --- | --- | --- | --- | --- | --- | --- | --- | --- |
|  |  | Unweighted UniFrac | | | | | | Bray Curtis | | |
| **Bacteria** |  | adonis / mantel | | | envfit / ordisurf | | | adonis / mantel | | |
| Group | Factor | R^2^ / r | P | | R^2^ | | P | R^2^ / r | P | |
| All samples (from Figure 1C) | Pitchers vs. environmental samples | **0.08** | **< 0.001** | | **0.31** | | **< 0.001** | **0.06** | **< 0.001** | |
|  | Location (HF, GC, S, and MB) | **0.09** | **< 0.001** | | **0.21** | | **< 0.001** | **0.09** | **< 0.001** | |
| *Nepenthes* samples | pH (range: 1.5 - 7.5) | **0.63** | **< 0.001** | | **0.74** | | **< 0.001** | **0.17** | **< 0.001** | |
|  | Volume (range: 0.01 - 500 mL) | 0.04 | 0.211 | | 0.07 | | 0.019 | -0.04 | 0.287 | |
|  | DNA concentration | 0.06 | 0.081 | | 0.03 | | 0.076 | 0.04 | 0.081 | |
|  | Pitcher species (8 species) | **0.18** | **< 0.001** | | **0.38** | | **< 0.001** | **0.15** | **< 0.001** | |
|  | Collection site (4 sites)* | **0.03** | **< 0.001** | | **0.09** | | **0.001** | **0.04** | **< 0.001** | |
| *N. gracilis* | pH (range: 1.5 - 6) | **0.46** | **< 0.001** | |  | |  |  |  | |
| *N. rafflesiana* | pH (range: 1.75 - 7) | **0.68** | **< 0.001** | |  | |  |  |  | |
| *N. stenophylla* | pH (range: 1.5 - 5.5) | **0.85** | **< 0.001** | |  | |  |  |  | |
| *Sarracenia* samples | pH (range: 3 - 8.5) | **0.11** | **0.010** | | **0.15** | | **< 0.001** | **0.07** | **0.010** | |
|  | Volume (range: 0.1 - 43 mL) | **0.15** | **0.006** | | **0.31** | | **< 0.001** | **0.17** | **< 0.001** | |
|  | DNA conc. (0.5 - 221 ng/uL) | **0.11** | **0.035** | | **0.22** | | **< 0.001** | 0.02 | 0.293 | |
|  | Pitcher species (6 species) | **0.14** | **< 0.001** | | **0.40** | | **< 0.001** | **0.17** | **< 0.001** | |
|  | Collection site (14 sites)* | **0.12** | **< 0.001** | | **0.31** | | **< 0.001** | **0.13** | **< 0.001** | |
| Natural *Nepenthes* and *Sarracenia* samples |  |  |  | |  | |  |  |  | |
|  | Genus | **0.09** | **< 0.001** | | **0.31** | | **< 0.001** | **0.08** | **< 0.001** | |
| Natural *Nepenthes* with experimental samples | pH | **0.50** | **< 0.001** | | **0.67** | | **< 0.001** | **0.30** | **< 0.001** | |
|  | Location (HF, GC, S, and MB) | **0.07** | **< 0.001** | | **0.19** | | **< 0.001** | **0.07** | **< 0.001** | |
|  | Pitchers vs. glass tubes | **0.02** | **< 0.001** | | **0.05** | | **< 0.001** | **0.02** | **< 0.001** | |
| **Eukaryotes** |  | adonis / mantel | | | envfit / ordisurf | | | adonis / mantel | | |
| Group | Factor | R^2^ / r | P | | R^2^ | | P | R^2^ / r | P | |
| All samples (from Figure 1C) | Pitchers vs. environmental samples | **0.08** | **< 0.001** | | **0.38** | | **< 0.001** | **0.03** | **< 0.001** | |
|  | Location (HF, GC, S, and MB) | **0.13** | **< 0.001** | | **0.28** | | **< 0.001** | **0.14** | **< 0.001** | |
| *Nepenthes* samples | pH (range: 1.5 - 7.5) | **0.14** | **< 0.001** | | **0.20** | | **< 0.001** | **0.06** | **0.050** | |
|  | Volume (range: 0.01 - 500 mL) | -0.04 | 0.809 | | **0.06** | | **0.023** | **0.10** | **0.006** | |
|  | DNA concentration | 0.02 | 0.349 | | 0.02 | | 0.131 | 0.04 | 0.171 | |
|  | Pitcher species (8 species) | **0.15** | **< 0.001** | | **0.22** | | **< 0.001** | **0.16** | **< 0.001** | |
|  | Collection site (4 sites)* | **0.03** | **< 0.001** | | **0.07** | | **0.008** | **0.03** | **< 0.001** | |
| *Sarracenia* samples | pH (range: 3 - 8.5) | -0.05 | 0.582 | | 0.02 | | 0.185 | -0.05 | 0.829 | |
|  | Volume (range: 0.1 - 43 mL) | **0.17** | **< 0.001** | | **0.18** | | **< 0.001** | **0.10** | **0.038** | |
|  | DNA concentration | 0.003 | 0.582 | | **0.08** | | **0.006** | -0.007 | 0.621 | |
|  | Pitcher species (6 species) | **0.20** | **< 0.001** | | **0.42** | | **< 0.001** | **0.31** | **< 0.001** | |
|  | Collection site (14 sites)* | **0.12** | **< 0.001** | | **0.31** | | **< 0.001** | **0.09** | **0.033** | |
| Natural *Nepenthes* and *Sarracenia* samples |  |  |  | |  | |  |  |  | |
|  | Genus | **0.14** | **< 0.001** | | **0.55** | | **< 0.001** | **0.15** | **< 0.001** | |
| Natural *Nepenthes* with experimental samples | pH | **0.11** | **0.001** | | **0.21** | | **< 0.001** | 0.03 | 0.069 | |
|  | Location (HF, GC, S, and MB) | **0.10** | **< 0.001** | | **0.41** | | **< 0.001** | **0.12** | **< 0.001** | |
|  | Pitchers vs. glass tubes | **0.03** | **< 0.001** | | **0.09** | | **< 0.001** | **0.02** | **< 0.001** | |
| Adonis and mantel tests are done on the dissimilarity matrices, while envfit and ordisurf are done on the NMDSs. Adonis and envfit are for categorical variables, while mantel and ordisurf are for continuous variables. P-values have been adjusted for multiple comparisons within each group using the Benjamini-Hochberg procedure. HF = Harvard Forest, GC = Gulf Coast, S = Singapore, MB = Maliau Basin. *In these calculations, the adonis test controls for pitcher species. | | | | | | | | | | |

**Table S5: Metagenomes for comparison with pitcher plants**

| Type | Source | ID |
| --- | --- | --- |
| lake | SRA | ERR358545 |
| lake | SRA | ERR358542 |
| lake | SRA | ERR358549 |
| lake | SRA | ERR358547 |
| phyllosphere | MG-RAST | 4447810.3 |
| phyllosphere | MG-RAST | 4447811.3 |
| phyllosphere | SRA | SRR2924445 |
| phyllosphere | MG-RAST | 4450328.3 |
| phyllosphere | MG-RAST | 4441205.3 |
| soil | MG-RAST | 4477899.3 |
| soil | MG-RAST | 4511045.3 |
| soil | MG-RAST | 4477804.3 |
| soil | MG-RAST | 4535147.3 |
| soil | MG-RAST | 4506447.3 |
| soil | MG-RAST | 4449357.3 |
| soil | MG-RAST | 4453436.3 |
| soil | MG-RAST | 4477807.3 |
| soil | MG-RAST | 4477875.3 |
| soil | MG-RAST | 4477874.3 |
