# Supplementary material for: Convergence between the microcosms of Southeast Asian and North American pitcher plants
Source: eLife. 2018 Aug 28;7:e36741. doi: 10.7554/eLife.36741 (PMC6130972; doi:10.7554/eLife.36741)
Supplement: Transparent reporting form [file elife-36741-transrepform.docx]

***eLife’s* transparent reporting form**

We encourage authors to provide detailed information *within their submission* to facilitate the interpretation and replication of experiments. Authors can upload supporting documentation to indicate the use of appropriate reporting guidelines for health-related research (see [EQUATOR Network](http://www.equator-network.org/%20)), life science research (see the [BioSharing Information Resource](https://biosharing.org/)), or the [ARRIVE guidelines](http://www.plosbiology.org/article/info:doi/10.1371/journal.pbio.1000412) for reporting work involving animal research. Where applicable, authors should refer to any relevant reporting standards documents in this form.

If you have any questions, please consult our Journal Policies and/or contact us: [editorial@elifesciences.org](mailto:editorial@elifesciences.org).

**Sample-size estimation**

- You should state whether an appropriate sample size was computed when the study was being designed
- You should state the statistical method of sample size computation and any required assumptions
- If no explicit power analysis was used, you should describe how you decided what sample (replicate) size (number) to use

Please outline where this information can be found within the submission (e.g., sections or figure legends), or explain why this information doesn’t apply to your submission:

We did not compute sample size before beginning the study. Our sample sizes were determined by fieldwork constraints – e.g. how many plants of each species were present, the restrictions of our collecting permits, and the amounts that we could carry and process while in field conditions. Numbers of different samples for each species are listed in Table S1 in the Supplementary Information.

**Replicates**

- You should report how often each experiment was performed
- You should include a definition of biological versus technical replication
- The data obtained should be provided and sufficient information should be provided to indicate the number of independent biological and/or technical replicates
- If you encountered any outliers, you should describe how these were handled
- Criteria for exclusion/inclusion of data should be clearly stated
- High-throughput sequence data should be uploaded before submission, with a private link for reviewers provided (these are available from both GEO and ArrayExpress)

Please outline where this information can be found within the submission (e.g., sections or figure legends), or explain why this information doesn’t apply to your submission:

Our experiment was performed only once due to restrictions of field conditions and resources. In comparisons of different species, each pitcher microcosm collected from a single species would be considered a biological replicate. A technical replicate would be the same pitcher microcosm sample that underwent separate DNA extraction and sequencing. We don’t have technical replicates for the study. We only excluded samples with fewer sequences than our stated cutoffs (listed in the paper, e.g. lines 582 and 595) or when there were multiples of the same sample (e.g. line 516 - 518) and we did not exclude outliers.

**Statistical reporting**

- Statistical analysis methods should be described and justified
- Raw data should be presented in figures whenever informative to do so (typically when N per group is less than 10)
- For each experiment, you should identify the statistical tests used, exact values of N, definitions of center, methods of multiple test correction, and dispersion and precision measures (e.g., mean, median, SD, SEM, confidence intervals; and, for the major substantive results, a measure of effect size (e.g., Pearson's r, Cohen's d)
- Report exact p-values wherever possible alongside the summary statistics and 95% confidence intervals. These should be reported for all key questions and not only when the p-value is less than 0.05.

Please outline where this information can be found within the submission (e.g., sections or figure legends), or explain why this information doesn’t apply to your submission:

This information can be found in sections “Analyses of 16S and 18S diversity” and “Functional analyses” on pages 27-30, and in Tables S1, S3 and S4.

(For large datasets, or papers with a very large number of statistical tests, you may upload a single table file with tests, Ns, etc., with reference to sections in the manuscript.)

**Group allocation**

- Indicate how samples were allocated into experimental groups (in the case of clinical studies, please specify allocation to treatment method); if randomization was used, please also state if restricted randomization was applied
- Indicate if masking was used during group allocation, data collection and/or data analysis

Please outline where this information can be found within the submission (e.g., sections or figure legends), or explain why this information doesn’t apply to your submission:

This information doesn’t apply to our study, as groups are defined by species identity.

**Additional data files (“source data”)**

- We encourage you to upload relevant additional data files, such as numerical data that are represented as a graph in a figure, or as a summary table
- Where provided, these should be in the most useful format, and they can be uploaded as “Source data” files linked to a main figure or table
- Include model definition files including the full list of parameters used
- Include code used for data analysis (e.g., R, MatLab)
- Avoid stating that data files are “available upon request”

Please indicate the figures or tables for which source data files have been provided:

The R source code and data are provided as supplementary material for Figures 1, 3, 4, 5 and their figure supplements, and for Tables S3 and S4. All additional files necessary to run the R code can be accessed from our Harvard Dataverse repository: [https://doi.org/10.7910/DVN/QYUBN2](https://doi.org/10.7910/DVN/QYUBN2). The Dataverse repository also has tree files and label files for the iToL trees in Figure 2 + supplement. Metagenomic data have been deposited with MG-RAST: <http://www.mg-rast.org/linkin.cgi?project=mgp15454>. Amplicon sequencing data have been deposited with the Sequence Read Archive as NCBI BioProject PRJNA448553.
